# Renin-Angiotensin System Blockade Associated with Statin Improves Endothelial Function in Diabetics

**DOI:** 10.5935/abc.20150123

**Published:** 2015-12

**Authors:** Ronaldo Altenburg Gismondi, Ricardo Bedirian, Cesar Romaro Pozzobon, Márcia Cristina Ladeira, Wille Oigman, Mário Fritsch Neves

**Affiliations:** Universidade do Estado do Rio de Janeiro, Rio de Janeiro, RJ - Brazil

**Keywords:** Hypertension, Renin-Angiotensin System/drug effects, Endothelium/physiopathology, Diabetes Mellitus

## Abstract

**Background:**

Studies suggest that statins have pleiotropic effects, such as reduction in blood pressure, and improvement in endothelial function and vascular stiffness.

**Objective:**

To analyze if prior statin use influences the effect of renin-angiotensin-aldosterone system inhibitors on blood pressure, endothelial function, and vascular stiffness.

**Methods:**

Patients with diabetes and hypertension with office systolic blood pressure ≥ 130 mmHg and/or diastolic blood pressure ≥ 80 mmHg had their antihypertensive medications replaced by amlodipine during 6 weeks. They were then randomized to either benazepril or losartan for 12 additional weeks while continuing on amlodipine. Blood pressure (assessed with ambulatory blood pressure monitoring), endothelial function (brachial artery flow-mediated dilation), and vascular stiffness (pulse wave velocity) were evaluated before and after the combined treatment. In this study, a post hoc analysis was performed to compare patients who were or were not on statins (SU and NSU groups, respectively).

**Results:**

The SU group presented a greater reduction in the 24-hour systolic blood pressure (from 134 to 122 mmHg, p = 0.007), and in the brachial artery flow-mediated dilation (from 6.5 to 10.9%, p = 0.003) when compared with the NSU group (from 137 to 128 mmHg, p = 0.362, and from 7.5 to 8.3%, p = 0.820). There was no statistically significant difference in pulse wave velocity (SU group: from 9.95 to 9.90 m/s, p = 0.650; NSU group: from 10.65 to 11.05 m/s, p = 0.586).

**Conclusion:**

Combined use of statins, amlodipine, and renin-angiotensin-aldosterone system inhibitors improves the antihypertensive response and endothelial function in patients with hypertension and diabetes.

## Introduction

Statins are the main LDL-cholesterol lowering drugs. Studies show that these drugs have pleiotropic effects, such as improvement in endothelial vasodilation response, increase in nitric oxide bioavailability, and reduction in endothelin levels^1,2^. These effects are believed to be related to their benefit in populations with high cardiovascular risk, in which their use is associated with reduced risk of cardiovascular events^3,4^.

One of the pleiotropic effects of the statins is the reduction in blood pressure (BP). A recent meta-analysis that included more than 20 thousand patients showed a reduction of 2.6 mmHg in systolic BP with statins^5^. In addition, this effect was greater in patients undergoing pharmacological treatment for hypertension, suggesting a potential synergistic mechanism between the antihypertensive drugs and the statins.

The aim of this study was to analyze whether the use of statin would influence the effect of renin-angiotensin-aldosterone system (RAAS) inhibitors on BP, endothelial function, and vascular stiffness in a population of patients with hypertension and type 2 diabetes mellitus (T2DM).

## Methods

### Patients Selection

Patients selected for this project had hypertension and T2DM and were followed up as outpatients in the internal medicine service at *Universidade do Estado do Rio de Janeiro (UERJ)*. The inclusion criteria were a previous diagnosis of hypertension and T2DM, age between 40 and 70 years, and systolic BP ≥ 130 mmHg and/or diastolic BP ≥ 80 mmHg. The exclusion criteria included resistant hypertension, insulin use, stages 4 and 5 chronic kidney disease, previous history of myocardial infarction and/or stroke, stages C and D heart failure, atrial fibrillation, symptomatic peripheral arterial disease, retinopathy with reduced visual acuity, nephrotic syndrome, and symptomatic diabetic neuropathy. The study was approved by the Research Ethics Committee of *Hospital Universitário Pedro Ernesto* (UERJ) under the number 01539612.6.0000.5259. All participants read and signed a free and informed consent form. The study is registered at ClinicalTrials.gov under the number NCT01603940.

### Study Design

The study was an open randomized clinical trial, with two active treatment groups and without use of placebo. At visit 1, patients underwent a clinical evaluation and had their antihypertensive medications replaced by amlodipine 5 mg/day. Visit 2 was conducted 6 weeks later and included clinical and laboratory evaluation, ambulatory BP monitoring (ABPM), and assessment of endothelial function and vascular stiffness. During this visit, the patients were randomized to benazepril 10 mg/day or losartan 50 mg/day while continuing on amlodipine. Two reevaluations (visits 3 and 4) were performed at intervals of 4 weeks for adjustment of BP, which was aimed at values < 130 x 80 mmHg. The antihypertensive medications in the study could have their doses doubled (benazepril 20 mg and losartan 100 mg), and the addition of hydrochlorothiazide 25 mg/day was allowed on visits 3 and 4. The dose of amlodipine was maintained at 5 mg/day. At the end of 12 weeks of treatment with RAAS inhibitors, the final visit (visit 5) was conducted, and the assessments performed on visit 2 were repeated. The dose of the T2DM medication was maintained constant. Adherence to treatment was assessed by a comparison between actual and expected tablet intake. We evaluated the use of antihypertensive drugs, statins, acetylsalicylic acid, and oral hypoglycemic agents. Patients were considered as having good adherence when the actual intake was equal to or greater than 80% of the expected intake.

### Statin Use

On visit 1, patients were classified as users or non-users of statin (SU and SNU groups, respectively). Throughout the 18 weeks of the study, the statin was maintained in those who were already taking it, and was not allowed to be started in those who were not taking it. The statin used in the study was simvastatin at a dose of 20 mg/day. All patients in the SU group were taking a statin for more than 12 weeks as prescribed by the physician assistant.

### Conditions to Undergo Complementary Evaluation

All tests were conducted in the morning after a 12-hour fast. Patients were instructed to not smoke, consume caffeine, or practice physical activity within 24 hours of the tests. The rooms were air-conditioned and had the temperature set to around 23 ± 2ºC and the air relative humidity between 50 and 70%. The patients were instructed to take the antihypertensive medication 1 to 2 hours prior to the evaluation.

### Blood Pressure Measurement

For office BP measurements, the patients sat down and rested for 10 minutes. We used a semiautomatic, calibrated equipment, model HEM-705CP (Omron Healthcare, Inc., IL, USA), with the cuff adjusted to the arm circumference. Three measurements were obtained in each arm, and their respective mean values were calculated. The highest mean value was used for analysis of the data.

### Ambulatory Blood Pressure Monitoring (ABPM)

ABPM was assessed with SpaceLabs 90207 (SpaceLabs Inc., WA, USA). The evaluation was scheduled to start between 8 and 9 a.m. and last for at least 24 hours. The BP was measured every 20 minutes between 6 a.m. and 11 p.m., and every 30 minutes between 11 p.m. and 6 a.m. The test was considered satisfactory when at least 70% of the readings were valid, with a minimum of 16 readings during the daytime and 8 readings during sleep and no more than two hours without measurements. We considered the sleep time as that reported by the patient in the activities diary. Nocturnal BP reduction was calculated as [(awake mean BP - sleep mean BP) /awake mean BP] x 100.

### Laboratory Tests

Venous blood samples were collected after a 12-hour fast for biochemical tests. When triglyceride levels were < 400 mg/dl, we calculated the LDL-cholesterol fraction with the Friedewald formula. Glomerular filtration rate (eGFR) was estimated with the MDRD formula: eGFR = 186*Creatinine^-1.154*^Age^-0.203^(*0.742 if female). C-reactive protein was determined by nephelometry (Siemens AG Inc., Munich, Germany). To measure microalbuminuria, we used the albuminuria/creatinuria ratio determined in a sample of first-morning urine.

### Vascular Tests

#### Brachial Artery Flow-Mediated Dilation (FMD)

The test was conducted according to the latest guidelines on the method^6^. The examiner was blinded to the patients’ treatment. We used the ultrasound equipment Vivid 3 (GE Healthcare, Milwaukee, WI, USA) with a 10 MHz high-resolution linear transducer. The patient was placed in the supine position, with the right arm abducted. After locating the brachial artery, the transducer was placed on the anteromedial surface of the right arm, perpendicular to the axis of the arm, 2 to 5 cm above the antecubital fossa, on the topography of the brachial artery. The measurements were obtained at the end of the diastole (R wave on the electrocardiogram). Ischemia was induced by inflation of the cuff 50 mmHg above the systolic BP during 5 minutes. The largest diameter of the artery was recorded 30, 60, and 90 seconds after deflation of the cuff. FMD was calculated as the variation of the largest diameter of the artery in relation to its baseline measurement.

#### Pulse Wave Velocity (PWV)

Pulse waves were obtained with the equipment Complior SP (Alam Medical, Paris, France). The transducers were positioned on the right carotid, right radial (crPWV), and right femoral (cfPWV) arteries^7^. The distances between the pulses were obtained directly with a tape measure. The carotid-femoral distance was multiplied by 0.8, and this value was used by the equipment for the calculation. Two PWV measurements were carried out, and when the difference between them was greater than 0.5 m/s, a third measurement was obtained. The average of these measurements was used for the analysis.

#### Determination of Central Aortic Pressures

Pulse waves at the right radial artery were obtained with a tonometer (SPC-301 - Millar Instruments, Texas, USA) calibrated according to the pressure in the brachial artery. Analysis of this arterial wave by applanation tonometry was performed to derive central aortic pressures and other hemodynamic parameters using the system SphygmoCor (Atcor Medical, Sydney, Australia)^8^. This software calculated the central systolic BP, central pulse pressure (PP), augmentation pressure (AP), and augmentation index (AIx). Two measurements were obtained, and when the difference between them was greater than 10%, a third measurement was performed. The average of these measurements was used for the analysis.

### Statistical Analysis

The data are presented as median (interquartile range) for numeric variables and distribution of frequencies and proportions for categorical variables. The Mann-Whitney test and Fisher's exact test were used to compare numerical and categorical variables, respectively. The Wilcoxon test was used for paired comparison of variables. Based on a maximum type I error of 0.05 and a standard deviation of 3.5%, a sample size of 14 patients in each group would have 80% power to find a difference ≥ 4% in FMD. The calculation of the sample size used the expected FMD difference between the benazepril and losartan groups according to the design of the original study^9^. The statistical analysis was performed using a significance level of 5% and the software Statistica 12 (Statsoft Inc, Tulsa, OK, USA).

## Results

**Figure 1:**
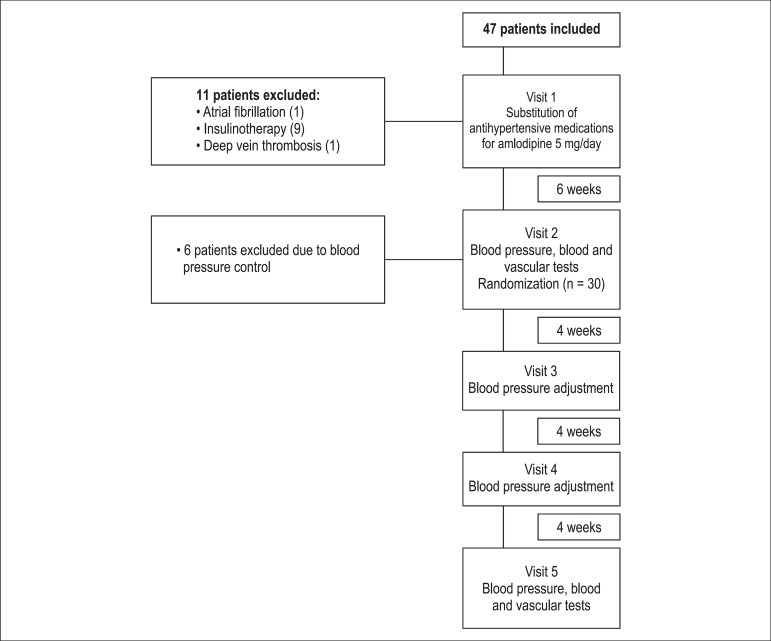
Flowchart of the participants throughout the study.

According to the inclusion criteria, 47 patients were selected, and 30 were randomized to one of the treatment groups (Figure 1). On visit 1, there were 13 patients in the SU group and 17 patients in the NSU group. All patients showed good treatment adherence. Baseline demographic, clinical, and laboratory characteristics of these patients are shown in Table 1. After treatment with the angiotensin-converting enzyme inhibitor (ACEI) or angiotensin receptor blocker (ARB), there was no statistically significant difference in laboratory parameters between the groups (data not shown).

**Table 1:** Baseline clinical and laboratory characteristics

**Variables**	**SU (n = 13)**	**NSU (n = 17)**	**p value**
Age (years)	58 (55-60)	57 (52-62)	0.401
Male Gender, n (%)	7 (53.8)	4 (23.5)	0.098
Smoking, n (%)	2 (15.3)	2 (11.7)	0.972
BMI (kg/m^2^)	29.4 (26.9-33.3)	31.2 (28.1-32.9)	0.999
Glucose (mg/dL)	107 (96-119)	117 (102-160)	0.438
HbA1c (%)	6.25 (5.80-6.70)	6.85 (6.30-7.60)	0.278
Creatinine (mg/dL)	0.76 (0.60-0.80)	0.70 (0.50-0.90)	0.899
eGFR (ml/min)	82.8 (77.8-105.7)	90.4 (72.6-130.8)	0.900
Potassium (mEq/l)	4.5 (4.3-4.7)	4.4 (4.0-4.7)	0.659
Uric Acid (mg/dL)	6.0 (4.5-6.1)	3.6 (3.2-5.1)	0.530
Triglycerides (mg/dL)	120 (95-174)	132 (102-162)	0.964
Total Cholesterol (mg/dL)	186 (167-218)	195 (171-217)	0.785
LDL-cholesterol (mg/dL)	106 (87-125)	117 (103-131)	0.645
HDL-cholesterol (mg/dL)	53 (49-56)	49 (44-59)	0.524
C-Reactive Protein (mg/dL)	0.18 (0.07-0.50)	0.48 (0.18-0.63)	0.089
ACR (mg/g)	16 (10-24)	14 (9-24)	0.747
Benazepril, n(%)	7 (53.8)	7 (41.1)	0.513
Metformin, n(%)	12 (92.3)	15 (88.2)	0.747
Sulphonylurea, n(%)	4 (30.7)	6 (35.3)	0.817

Values are expressed as median (interquartile range), except where specified otherwise; SU: Statin users group; NSU: Non-statin users group; BMI: Body mass index; HbA1c: Glycated hemoglobin; eGFR: Estimated glomerular filtration rate; LDL: Low-density lipoprotein; HDL: High-density lipoprotein; ACR: Albumin-creatinine ratio.

### Blood Pressure

**Figure 2:**
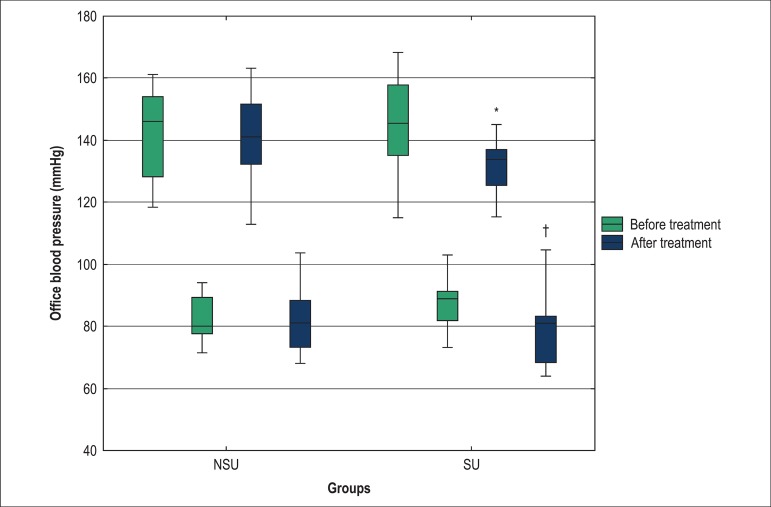
Comparison of office systolic and diastolic blood pressure values between the SU and NSU groups before and after association of antihypertensive treatment; SU: Statin users group; NSU: Non-statin users group; *p = 0.100, patients in the SU group before and after treatment; †p = 0.016, patients in the SU group before and after treatment.

**Figure 3:**
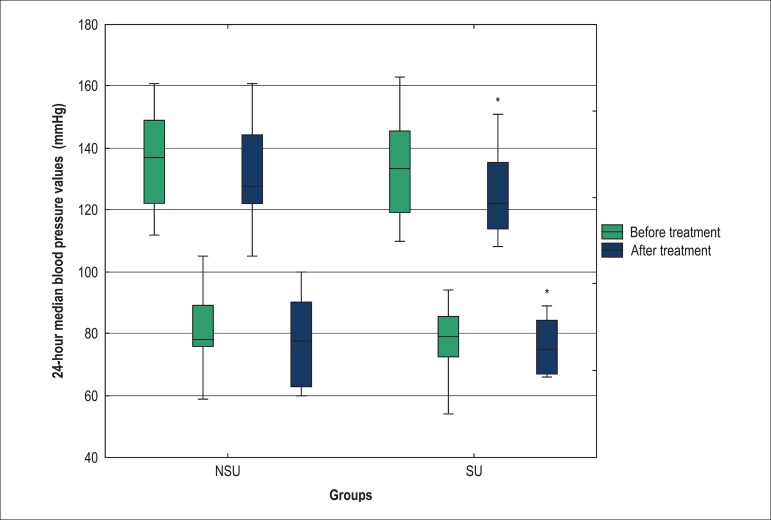
Comparison of mean 24-hour systolic and diastolic blood pressures in the SU and NSU groups before and after association of antihypertensive treatment; SU: Statin users group; NSU: Non-statin users group; *p = 0.007, patients in the SU group before and after treatment.

In the analysis of the office BP in the SU group, there was a non-significant decrease in diastolic (8.0 mmHg or 9% reduction) and systolic BP (11.6 mmHg or 8% reduction), although no statistical significance was observed (Figure 2). In the ABPM analysis, the SU group exhibited a greater reduction in mean 24-hour systolic BP (11.5 mmHg or 9% reduction) (Figure 3 and Table 2). A similar effect was observed in the mean 24-hour diastolic BP (4.0 mmHg or 5% reduction) and PP (7.0 mmHg or 13% reduction) (Table 2).

**Table 2:** Effect of statin use on ABPM.

**Variables**	**SU**		**NSU**		**p value**		
	**Before**	**After**	**Before**	**After**	**p1**	**p2**	**p3**
SBP 24-h ABPM	134 (120-146)	122 (114-135)	137 (122-149)	128 (122-140)	0.007	0.362	0.333
DBP 24-h ABPM	79 (73-86)	75 (67-84)	78 (76-89)	78 (63-90)	0.007	0.209	0.976
PP 24-h ABPM	56 (49-61)	49 (41-55)	51 (45-62)	56 (44-61)	0.032	0.813	0.177
Awake SBP	139 (121-149)	125 (115-136)	139 (125-156)	130 (123-147)	0.015	0.167	0.333
Awake DBP	81 (74-89)	77 (72-83)	81 (78-92)	80 (66-90)	0.058	0.099	0.930
Nocturnal SBP	125 (112-139)	113 (109-119)	134 (116-137)	121 (109-132)	0.035	0.396	0.278
Nocturnal DBP	72 (67-76)	67 (58-73)	73 (66-83)	67 (62-80)	0.035	0.615	0.769
Nocturnal Descent (%)	5.0 (2.2-11.7)	7.4 (2.9-11.5)	10.2 (4.3-13.3)	6.4 (4.6-8.2)	0.374	0.432	0.578

Values are expressed as median (interquartile range), except where specified otherwise; unit: mmHg; p1: Patients in the SU group before and after treatment; p2: Patients in the NSU group before and after treatment; p3: Comparison between the SU and NSU groups at the end of the treatment; SU: Statin users group; NSU: Non-statin users group; SBP: Systolic blood pressure; DBP: Diastolic blood pressure; PP: Pulse pressure; ABPM: Ambulatory blood pressure monitoring.

There was a similar percentage of patients using ACEI (54 vs 41%) and ARB (46 vs 59%) in the SU and SNU groups, and a similar proportion of patients who required dosage adjustment of these medications (61% vs 47%, p = 0.430) and/or association of hydrochlorothiazide for BP control (15% vs 17%, p = 0.865).

### Endothelial Function and Vascular Stiffness

**Figure 4:**
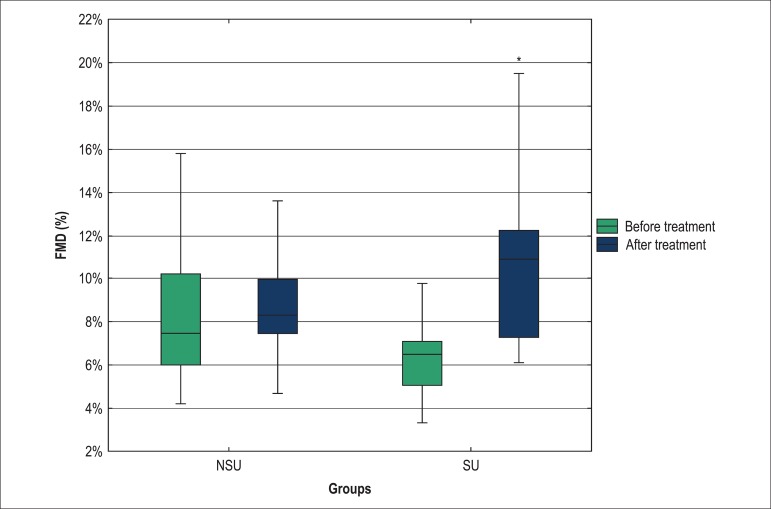
Comparison of the values of brachial artery flow-mediated dilation between the SU and NSU groups before and after association of antihypertensive treatment; SU: Statin users group; FMD: Brachial artery flow-mediated dilation; NSU: Non-statin users group; *p = 0.003, patients in the SU group before and after treatment.

The SU group presented a greater FMD response and a higher reduction in systolic aortic BP when compared with the NSU group (Figure 4). There was no statistically significant difference in AIx, cfPWV, and crPWV responses (Table 3).

**Table 3:** Effect of statin use vascular tests

**Variables**	**SU**		**NSU**		**p value**		
	**Before**	**After**	**Before**	**After**	**p 1**	**p 2**	**p 3**
Brachial FMD (%)	6.50 (5.10-7.10)	10.90 (7.30-12.20)	7.50 (6.00-10.20)	8.30 (7.50-9.95)	0.003	0.820	0.211
cfPWV (m/s)	9.95 (9.35-10.55)	9.90 (8.50-11.05)	10.65 (9.60-12.35)	11.05 (10.20-11.70)	0.650	0.586	0.111
crPWV (m/s)	9.85 (9.05-10.40)	10.00 (9.05-10.55)	9.70 (9.35-10.90)	9.65 (9.35-10.20)	0.972	0.570	0.490
Aortic systolic BP (mmHg)	139 (128-143)	125 (117-128)	127 (121-140)	126 (119-139)	0.046	0.623	0.477
Aortic PP (mmHg)	86 (84-92)	82 (68-84)	82 (79-87)	79 (74-88)	0.018	0.508	0.706
AIx (%)	30 (28-43)	33 (25-40)	31 (23-33)	33 (27-37)	0.263	0.308	0.916
AP (mmHg)	15 (13-24)	17 (10-21)	13 (9-18)	16 (10-20)	0.278	0.320	0.966

Values are expressed as median (interquartile range), except where specified otherwise; p1: Patients in the SU group before and after treatment; p2: Patients in the NSU group before and after treatment; p3: Comparison between the SU and NSU groups at the end of the treatment; SU: Statin users group; NSU: Non-statin users group; Brachial FMD: Brachial artery flow-mediated dilation; cfPWV: Carotid-femoral pulse wave velocity; crPWV: Carotid-radial pulse wave velocity; BP: Blood pressure; PP: Pulse pressure; AIx: Augmentation index; AP: Augmentation pressure.

## Discussion

The most recent guidelines recommend that patients with T2DM should use RAAS inhibitors (ACEI or ARB) and statins^10-13^. The present study showed that simvastatin improves endothelial function and increases the antihypertensive effect of ACEI or ARB in association with amlodipine in patients with hypertension and diabetes.

The antihypertensive mechanisms of the statins are probably due to an increase in nitric oxide bioavailability with an increase in endothelium-dependent vasodilation response, and a reduction in endothelin-1 concentration and free radicals formation^1,2,14^. In addition, a synergism is also speculated between statins and RAAS inhibitors through reduced expression of type 1 angiotensin receptors and blockade of intracellular pathways associated with angiotensin II action, both caused by statins^15^. Many of these benefiting mechanisms of the statins are independent of the reduction in LDL-cholesterol. In the present study, the reduction in BP and improvement in endothelial function occurred despite statistically similar LDL-cholesterol values in the SU and NSU groups.

In a recent meta-analysis, Briasoulis et al.^5^ observed a mean systolic BP reduction of 2.6 mmHg in patients on statins, even in those without a diagnosis of hypertension^5^. In hypertensive patients, the systolic BP may reduce up to 5.8 mmHg^5^. In diabetics, the study estimated a reduction of 6.5 mmHg in the systolic BP and 4.0 mmHg in the diastolic BP^5^. Another meta-analysis by Strazzullo et al. also examined the antihypertensive effect of statins and observed an average reduction of 4.0 mmHg in the systolic and 1.2 mmHg in the diastolic BP^16^. Strazzullo et al^16^ observed in a subgroup of diabetic patients a reduction of 3.7 mmHg in the systolic and 0.8 mmHg in the diastolic BP^16^. None of these studies investigated the association of statins with ACEI or ARB.

Spósito et al^17^ studied the interaction between ACEI and statins and observed that the group receiving a combination of both showed greater BP reduction when compared with the group receiving ACEI alone (21 vs. 14 mmHg, p < 0.05)^17^. However, Mancia et al^18^ observed no reduction in BP with the association of pravastatin and fosinopril. Koh et al^19^ evaluated the combination of losartan and ramipril with simvastatin and did not observe greater BP reduction with this association^19,20^. In the present study, the association of benazepril or losartan in patients already receiving simvastatin and amlodipine promoted greater reduction in systolic and diastolic BP, both in casual measurements in the office and in 24-hour measurements.

Zhang et al^21^ recently published a meta-analysis on the effects of statins in brachial artery FMD in patients with T2DM. The final result showed an improvement of 0.94% (95% CI 0.38-1.50%, p < 0.001) in absolute FMD values. Koh et al^19^ conducted a study in which patients were randomized to three groups: ramipril, simvastatin, or a combination of both. Their cohort, comprised of 76% of hypertensive patients, included individuals with a mean age of 60 years, T2DM, and LDL-cholesterol > 100 mg/dL. The authors observed that the combined use of statins and ACEI promoted a greater increase in FMD than each medication alone. In contrast, no improvement in FMD was observed by Van Venrooij et al^22^ with atorvastatin, and Beishizen et al. with cerivastatin and simvastatin^22,23^. Tan et al^24^, in a population with T2DM and dyslipidemia, observed an improvement in FMD with atorvastatin when compared with placebo. In the present study, which assessed a population of diabetic patients with hypertension, only those already using simvastatin had a statistically significant improvement in brachial artery FMD when benazepril or losartan were associated.

Kanaki et al^25^ evaluated the use of simvastatin in patients with hypertension and dyslipidemia and observed improvements in PWV, aortic BP, and AIx. However, no improvement in aortic pressure was observed with atorvastatin by William et al^26^ in hypertensive patients and Fasset et al^27^ in patients with chronic renal failure^26,27^. Raisone et al^28^ were one of the few to observe worsening in PWV with atorvastatin. In contrast, Pirro et al^29^ and Maki-Petaja et al^30^ observed improvement in PWV with statins. So far, there are no studies on the effects of statins on aortic pressure and PWV in diabetic patients with hypertension. The present study showed a reduction in central aortic pressure, in parallel with reductions in office BP and ABPM in simvastatin users, although no statistically significant changes were observed in AIx and PWV.

The study has some limitations. Since this is a *post hoc* analysis, the groups were not randomized according to statin use. In addition, approximately half of the study sample had no prior use of statin. Although it was not possible to evaluate the reasons, the group without prior use of simvastatin could have pre-selected patients with poor adherence to antihypertensive drugs before recruitment for the study. These patients could have BP levels above the recommended target, higher prevalence of vascular lesions, and as a result, inadequate response to antihypertensive drugs or lower improvement in endothelial function with the treatment when compared with the group of statin users. There was also a trend towards more male patients in the group of simvastatin users. We believe that this fact had little relevance, because despite having a higher cardiovascular risk, this group had the greatest benefit in BP reduction. The open use of the medication is also a limiting factor. However, the results were reinforced by the BP reduction in the ABPM and in central aortic measurements, since these methods are known to be minimally influenced by the placebo effect.

## Conclusions

Prior use of statin increases the antihypertensive effect and improves the endothelial function in hypertensive patients with T2DM treated with amlodipine associated with an RAAS inhibitor. Randomized studies with larger samples and 2x2 design are needed to evaluate the interaction between statins and RAAS inhibitors.
